# Measuring the Impact of Virtual Communities on the Intention to Use Telemedicine Services

**DOI:** 10.3390/healthcare10091685

**Published:** 2022-09-04

**Authors:** Iustin Priescu, Ionica Oncioiu

**Affiliations:** 1 Department of Informatics, Faculty of Informatics, Titu Maiorescu University, 040051 Bucharest, Romania; 2 Faculty of Finance-Banking, Accountancy and Business Administration, Titu Maiorescu University, 040051 Bucharest, Romania; 3 Doctoral School of Economic Sciences, University of Craiova, 200585 Craiova, Romania

**Keywords:** digital marketing, telemedicine, promotion strategies, social media platforms, medical institution

## Abstract

Digital marketing has given new life to healthcare services by enhancing their visibility in the online space. People choose online healthcare services because they can receive instant answers and communicate with specialists in their comfortable environment at the right time. The purpose of this study was to understand the impact of virtual communities on the intention to use telemedicine. The model is based on a combination of consumer desire (psychological objective) and loyalty through promotional formats (economic objective), as well as data collected from 442 respondents analyzed using structural equation modeling. The research results show that by analyzing target groups in social networks, content can be individualized, and an accurate measurement of e-patient satisfaction must be conducted in order to improve the experience of future consumers of telemedicine services. The results of this study explain what makes people want to use digital healthcare services and can serve as a guide for people who run virtual communities and help digital healthcare service providers figure out how to market their services.

## 1. Introduction

Information technology in the health sector aims to streamline processes, increase patient safety, create new forms of care, and improve health [1,2,3]. In this context, interest in telemedicine has increased significantly, reaching over 30% of private medical activity [4]. Although it is considered that telemedicine will not become an absolute substitute for physical consultations, it remains extremely useful for both doctors and patients in cases where it is not necessary to be present in the office and thus favors unrestricted consultations despite geographic barriers [5]. However, because there is more competition in the field, technology is getting better, and people’s lives are changing, digital marketing is being used more and more in the field of telemedicine [6,7,8].

On top of that, telemedicine solutions allow the resolution of time and space, i.e., a diagnosis can be made, or therapy can be evaluated without the health professional and the patient being in the same room at the same time [4]. Although direct contact with patients is undeniably important in caregiving, telemedicine care can supplement treatment and alleviate possibly long travel or wait times [5]. From a medical point of view, this kind of treatment can even improve the quality of care because it can be much more attentive (for example, by keeping an eye on vital signs) than treatment based on personal contact alone [8].

Especially during the COVID-19 pandemic period, the health system changed radically as a result of digitization processes and produced not only new products, but also new service practices and business models, replacing conventional structures [9]. According to e-patient characteristics, telehealth can be categorized as follows: (1) interventions for online patients (targeting current or potential medical service recipients, including caregivers); (2) interventions for telemedicine service providers (targeting medical staff); (3) interventions for e-health systems or resource management (targeting managerial resources used in the administration and supervision of public health services); and (4) interventions for data collection, management, and the use of online services [4,10]. 

The implementation of integrated care models also improves the efficiency and responsiveness of e-health systems [7]. According to this view, new technologies must neither aggravate socioeconomic inequities nor impair public health objectives while maintaining and respecting essential values [11]. Utilizing digital services will be necessary in order to accomplish this objective; these include portable devices, mobile health applications, and mobile communication devices for health, welfare, and informational purposes [12].

Advanced digital technologies also improve national health systems by expanding the scope, transparency, and accessibility of online healthcare services and information, and by improving the provision of services to patients [4,13]. Telehealth plays an essential role in achieving strategic public health priorities because it reduces risk factors and health determinants by supporting intersectoral mechanisms, and it promotes the reduction in e-healthcare disparities and enhanced well-being [14,15,16]. The aims of sustainable development and digital solutions may be used to strengthen efforts to attain universal telehealth coverage, as well as disrupting and reforming the underlying processes of e-health systems [17].

Moreover, digital marketing is under constant pressure to contribute as much as possible to business success [11]. Telemedicine patients in virtual communities can be processed through self-learning systems, which are autonomous and decisive [12]. Thus, digital media is expanding the existing contact points and giving companies that offer this type of service better access to patients through telemedicine [18]. The significance of digital marketing is unquestionably connected to the modification and standardization of the marketing mix in accordance with the characteristics of target online patients for the services being offered [13]. To that end, several marketing mix options might be prioritized: e-healthcare services, promotional communication, and pricing [19].

In this communication process, more rating portal operators appear as intermediaries [20]. Patients’ ratings on rating portals must be accepted by physicians and clinics without their consent, or even against their expressly stated wishes. The basic problem remains the unrestricted protection of anonymous opinions, which means that doctors cannot regularly claim their criticism, and portal operators act as intermediaries in this asymmetric communication process [21]. From the user’s point of view, the special value of a portal is that it receives ratings from other users who have already used medical or clinical services [20]. In general, review portals can help protect patients online by giving them information and, as a result, making the market transparent [22].

Supporting this view, our paper takes a fresh look at empirical studies that have attempted to demonstrate the link between organizational performance and digital marketing in Romanian telehealth companies. Based on a sample of 442 respondents (108 social media managers of digital healthcare service providers, 20 influencers specializing in e-health, and 314 online patients) who had experience using telemedicine services on social media, the collected data were analyzed using structural equation modeling. The authors combined constructs (promotional format, number of recommendations, perceived credibility, social influence, and information quality) to determine the impact of virtual communities on overall telehealth usage intent. In all of the scientific processes involved, these constructs are considered to be useful in answering the following research questions: What is the impact of social media on the intention to use telemedicine services? Do promotional formats affect patients’ intention to use medical services through social networks? Do the information search behavior and the intention to use telemedicine services vary depending on the number of recommendations?

According to the research results, a coherent and efficient solution to the problems faced by Romanian healthcare companies will be conditioned by actions aimed at segmenting online patients based on behavior, the use of online reviews, or the identification of unusual behavior. Of course, Romanian managers need to adapt, think internationally, and become more competitive by using the digital environment. The obtained findings are also valid and reproducible because they highlight patients’ behavior intention when selecting service providers under the pressure and influence of the “online herd”.

Moreover, this study conceptualizes digital marketing from the perspective of companies that provide telemedicine in Romania by establishing cause–effect relationships and demonstrating the validity of the variables analyzed. Building credibility and relevance (as well as customer loyalty) is an additional digital marketing priority for these companies. This study provides a foundation for future research on e-healthcare users’ motives, which may be used to expand the reach of online communities and inform specific marketing strategies.

Unlike other studies that focused on only a few dimensions [20,21,22], we propose a multidimensional approach to understanding information-seeking behavior and decision-making regarding the intent to use digital healthcare services. This contributes to the existing body of knowledge on the relationship between online patients, influencers, and digital healthcare service providers by establishing the main factors that encourage people to get involved in the digital environment when they intend to use or recommend a medical service. Thus, digital processing and the exchange of patient and health data offer enormous innovation potential for healthcare providers toward better quality and more efficient services.

The rest of the paper is organized as follows: the literature review and hypothesis formulation are presented in Section 2; the research model is presented in Section 3; the empirical results and implications for discussion are described in the following sections; and the conclusions are presented in the last section. 

## 2. Literature Review and Hypothesis Development

### 2.1. Telemedicine: Innovative or Complex Solution

Digitization, big data analysis, and smart data have reached the healthcare industry [23]. The digital healthcare industry is increasingly affected by factors such as the data life cycle, digitization, definitions, and the development of big data for long-term healthcare. In the literature, this phenomenon, known as telemedicine, is extremely multifaceted in terms of its technological, legal, and economic aspects and is both a challenge and an opportunity for private and public medical institutions [24,25,26]. With the increase in digitalization, the role of the patient has changed to that of an autonomous and informed e-patient, which means that the structures of the e-health system must continually adapt to the preferences of online patients [27].

Today’s patient is referred to in the recent literature as an e-patient [28,29,30]. This refers to a well-informed patient who exchanges information with others on online platforms, actively participates in decisions, and awaits further requests to participate in their own healthcare. Digital networks offer a new dimension of availability of information and therefore offer online patients the opportunity to expand their knowledge independently. According to previous studies [31,32], the central requirements for patients are to improve the framework conditions, expand the infrastructure at the technical and legal level, eliminate gaps in information and knowledge, and ensure data security.

Evaluation portals, therefore, provide not only a market for opinions but also forums for the unilateral criticism of telemedicine services [28]. The communication process in virtual communities is characterized by asymmetry, as the patient, protected by anonymity, is allowed to make any statement, which is immediately published by the portal operator without any initial control. Since evaluations can be sent and posted at any time, doctors and hospitals are, in theory, forced to always keep an eye on their digital presence. Patients are also drivers of digitization in the healthcare system, which is why we need to work quickly to find appropriate solutions to e-patients’ needs [3].

Another opportunity related to the use of telemedicine is reduced cost, as a result of efficiency gains for both patients and the state or payers [33]. On the one hand, cost reduction is possible because unnecessary double examinations are avoided, and better prevention is achieved with the help of remote diagnosis and optimized information processing. On the other hand, there is the potential to reduce waiting times by providing electronic prescriptions and using telemedicine consultation schedules.

On that basis, the promotion of e-healthcare services can take on various forms, particularly in terms of the selection of consumers to whom the services are oriented [16,17,18,19]. The purpose of promotion by any health institution is to interact with past, present, and potential e-patients in order to make them aware of the available digital healthcare services. As a result of the digital transformation process and the rise of interactions in the online environment, social media platforms provide channels of information for the healthcare system and the promotion of digital healthcare services, therefore contributing to the development of e-health [33].

### 2.2. Theoretical Framework

Research investigating the impact of virtual communities on the intention to use telemedicine has been gaining attention in recent years. Basically, it can be said that changes in the behavior of people using online medical services were primarily triggered by technological innovations. The original analysis focused on psychology and sociology rather than acceptance and technology, but lately the attention has shifted to technology acceptance and use, with the technology acceptance model (TAM) being increasingly used [34]. Venkatesh et al. [35] developed the unified theory of acceptance and the use-of-technology (UTAUT) model based on the TAM by synthesizing existing technology acceptance research. The UTAUT factors (performance expectation, effort expectation, social influence, and enabling conditions) have mainly been used to predict behavioral intention to use technology [9].

In addition, in order to persuade consumers to use telemedicine, it is essential to identify and understand the barriers that cause resistance to its adoption as well as the drivers that contribute to it. Several theories and models of information systems (IS) provide a big-picture view on how to adopt and use technological innovations, which is what this research area is about. The IS success model hypothesizes that information quality is an essential component influencing user satisfaction and usage, and that these are important variables in determining net benefits. A literature review [36] noted that the IS success model has been combined with different theories to investigate user intention and information system behavior. For example, the IS success model can be combined with TAM to describe users’ intention to use and satisfaction with e-health services. Our study, which is within this context, is intended to improve the explanatory capacity of the perception of e-patients by developing the frameworks presented above that combine psychological and economic objectives.

This study proposes a combination of frameworks—the TAM, UTAUT, and IS success models—to analyze the impact of virtual communities on the intention to use telemedicine. We used these models as a theoretical foundation to develop a patient-centered research paradigm for online healthcare that highlights the importance of patient preferences.

### 2.3. Information Quality

The landscape of telemedicine services is much more diverse than is commonly assumed, and the great heterogeneity in terms of the accessibility, depth, and breadth of information makes it difficult to form a comprehensive view. Information quality has a critical role in building user satisfaction with and e-patient loyalty to digital healthcare services [37]. In addition, it is essential to have comprehensive data for the purpose of providing not only high-quality clinical treatment, but also ongoing healthcare. Studies have shown that more and more e-patients are interested in finding and evaluating health information, having their personal health data protected where necessary, evaluating the functionality, outcomes, and consequences of digital health applications, and weighing the advantages and disadvantages [37,38,39]. For all that, the quality of the information is still limited because the primary focus is on analyzing and providing partial solutions following the introduction of e-health applications. The analysis of the completeness of e-healthcare service information refers to the extent to which all possible conditions relevant to users are available in the stored information. In terms of updating, this is the way to improve the quality of the information. As such, the quality of information could affect the behavioral intent of e-patients toward telemedicine services.

### 2.4. Use Intention

From a literature perspective, the intention to use can be defined as a subjective belief that leads to certain behavior in the future [40]. Studies have shown that information quality is an important aspect that generates exchanges of views among community members [41,42]. Telemedicine services and recommendation data can be provided through virtual communities, where members interact, exchange perspectives, and customize the information. Users can share their activity with others, and the information is displayed on the Web page [43,44]. Network features allow those who stay at home to connect to the rest of the world or from a variety of databases. Based on the previous studies, the following hypothesis was proposed:

**Hypothesis** **1** **(H1).** *Information quality positively influences the intention to use telemedicine services.*

### 2.5. Promotional Formats

Firms often implement new promotion strategies by adopting various promotional formats to encourage patients to use digital medical services [45]. According to several studies, promotional formats for online healthcare services can be divided into monetary promotions (such as price reductions) and non-monetary promotions (such as bonuses and bonus packages) [46,47,48]. Monetary promotions mainly offer money-saving benefits, while non-monetary promotions offer hedonistic benefits to incentive-oriented consumers without comparing telemedicine service prices. Therefore, consumers will be stimulated by the price reduction based on the amount of savings perceived when deciding to use digital healthcare services [48]. Furthermore, some authors [46,49] have found that when companies offer both premium promotions and price reductions equally, consumers will prefer price reductions, which leads to a higher intention to use digital healthcare services. Based on non-monetary promotions, in this study, we used bonus packages to investigate the behavior of online patients with regard to digital marketing, focusing on the information quality related to telemedicine services promotions. Therefore, the following hypothesis was proposed:

**Hypothesis** **2** **(H2).** *Promotional formats via social media significantly affect the* *quality of information regarding telemedicine services.*

In other words, web users have greater opportunities and flexibility when choosing digital healthcare services because they can make good use of the media feature to control their own time and the amount of information, while also having the opportunity to break social norms [50]. Thus, doing away with practical controls and regulations allows customers the freedom to make informed decisions when selecting e-healthcare providers [44]. In order to incorporate conversation, members of the network can express their feelings and opinions regarding various advertising formats using the functionalities of web pages and blogs, for example [43]. Therefore, this study also included promotional formats among the motivations that affect the intention to use telemedicine services. The following is the third hypothesis of this study:

**Hypothesis** **3** **(H3).** *The more positive social media users’ feelings are about promotional formats, the greater their intention to use telemedicine services.*

### 2.6. Number of Recommendations

Some providers of digital healthcare strategically handle online reviews in an effort to influence patients’ usage decisions. Studies have shown that e-patients are strongly influenced by the information they deduce from the behavior of others (for example, the number of downloads) [44,51,52]. Research evidence has suggested that user recommendations are critical in e-patients’ decisions about using e-healthcare services [27]. Sometimes, users of digital healthcare services believe that their findings are not the best ones possible because they do not engage in more extensive searches that require more time to browse the web [50]. However, the situation is different for the number of recommendations, because people can find information easily, quickly, and accurately. Moreover, the more recommendations that are available, the greater the effect they have on e-patients’ behavior [52]. Even with a small number of suggestions, a member without persuasive points will not be recognized as knowledgeable [53]. Thus, this study proposed the following hypothesis:

**Hypothesis** **4** **(H4).** *In virtual communities, the number of recommendations has a positive effect on the information quality regarding telemedicine services.*

The first condition for success in digital marketing is that digital healthcare services have an indisputable quality, thus they promote themselves [53]. Beyond that, some digital healthcare companies use a referral process that is substantially affected by the introduction of incentives, such as commissions or rewards for those who make recommendations [50]. Online patients are actively looking for recommendations, which can be the key differentiator between similar telemedicine services. Statistics have shown that social proof of recommendation marketing (or proof that others use telemedicine services and are excited) will become indispensable for referral marketing strategies [15]. Therefore, this study included the number of recommendations among the motivations that affect the intention to use telemedicine services. As a result, the following hypothesis was proposed:

**Hypothesis** **5** **(H5).** *The number of recommendations has a significant influence on the* *intention to use telemedicine services.*

### 2.7. Perceived Credibility

Perceived credibility in virtual telemedicine communities refers to how confident patients feel that their digital healthcare provider has the knowledge to address their health concerns [26,54]. If patients believe that the healthcare service provider they are using online is not trustworthy, they will not trust the service being offered. Discussion forums, news blogs, and review websites are examples of neutral online sources that can contribute to building trust [55]. More than just an outcome, credibility can be characterized as a psychological condition that includes the intention to recognize vulnerability based on favorable expectations for the behavior of others. This component was examined by several researchers [54,55,56], who discovered a substantial positive association between credibility and the quality of information offered, along with a favorable influence on the intention to use e-healthcare services. Moreover, some researchers [57,58,59] have proposed that patients’ trust in digital healthcare services is influenced by their perception of the reliability of those services. Hence, the following hypotheses were proposed:

**Hypothesis** **6** **(H6).** *Perceived credibility has a significant influence on the* *quality of information regarding telemedicine services.*

**Hypothesis** **7** **(H7).** *Perceived credibility significantly affects the intention to use telemedicine services.*

### 2.8. Social Influence

The idea behind social influence in the context of telehealth is that while an individual may not be in favor of using an online medical service, they may intend to share or comment on it because they believe it will enhance their image among family members and colleagues [57]. Social media provides a wealth of information on digital healthcare services in addition to developing and maintaining social relationships [60,61,62]. The demand for inclusion comes from a desire to be part of the group, but the demand for control comes from a desire to successfully influence matters, people, and things. Individual differences exist in people’s need for control. The ability to perform a social role is improved by an acceptable need for control, because individuals who have this characteristic would avoid responsibility and lack the will to exert influence over their circumstances. On the other hand, influencers also have an important role to play in assuring the quality of information about digital healthcare services; they work well as an alternative to traditional advertising and give the company more credibility [63,64,65,66,67]. Therefore, companies have an opportunity to disseminate and publish content directly through influencers in a fair and legal framework, and in a credible and authentic way. Thus, content can be communicated directly to target groups without wasting any time, through influencers who specialize in specific telemedicine services and assist in delivering proper information to allow followers to make educated decisions. Additionally, the advantage of blog posts published by influencers is that the content can be found over an extended period of time. So, influencers are an integral part of the research process since they can educate patients and help them make well-informed decisions. Thus, the following hypothesis was proposed.

**Hypothesis** **8** **(H8).** *Social influence has a positive effect on the* *quality of information regarding telemedicine services.*

It should also be noted that the inseparability of individual behavior from social influence is defined as a social relationship [67]. Compliance refers to private activities being in accordance with society’s norms. Previous studies [67,68,69,70] noted that the norm is the objective standard of conduct in the name of public opinion, and compliance comes from the individual’s psychological tendencies. Despite this, pressure from the group will have an effect on people’s decision making [71]. Based on this discussion, this study includes social influence as one of the factors with an impact on the intention to use telemedicine services. The hypothesis was proposed as follows.

**Hypothesis** **9** **(H9).** *Social influence significantly influences the intention to use telemedicine services.*

## 3. Research Methodology

**Figure 1:**
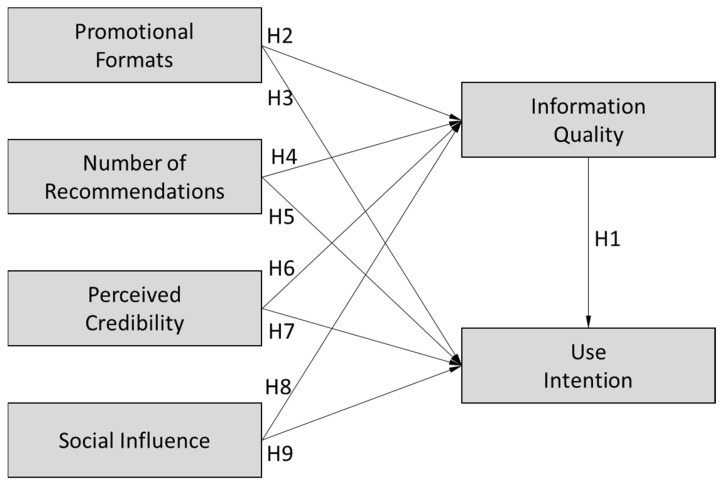
Proposed research model.

This study examined the effect of four variables on the interaction quality of social media users: promotional forms, number of recommendations, perceived credibility, and social influence. In addition, we examined the relationship between information quality and the intent to use telemedicine services. Figure 1 illustrates the proposed research model.

To ensure the validity of the research, the questions were based on previous relevant studies and were professionally updated and appropriately adjusted for the context of this study, as listed in Table 1. The scales for the constructs were adapted from DeLone and McLean [37], Venkatesh and Zhang [72], and Nisha et al. [73]. 

The study involved an anonymous online survey and was conducted between February and May 2022. A 5-point Likert scale, ranging from 1, strongly disagree, to 5, strongly agree, was used in the survey questions. The draft questionnaire was pre-tested with 20 online health influencers to ensure that the questions were as fluent and expressive as possible. 

The use of the snowball sampling method allows authors to avoid biases in data and provides equal opportunities for all respondents who may be potential users of telemedicine services. The questionnaires were completed by 442 respondents, representing a response rate of 94%. The link for the survey was sent to 108 responsible social media managers within each telemedicine service provider in the sample, and they sent the link to 314 e-patients and 20 influencers who specialize in e-health, selected through their own media channels in accordance with the data presented in Table 2. 

The analysis was performed using structural equation modeling. The measurement model was first examined to assess the reliability and validity of the constructs, and then the structural model was analyzed to determine the effects of dependent and independent variables using path model analysis. It was concluded that the reliability of the framing measurement was appropriate to test the hypotheses.

## 4. Results

Using the indicators described above, we assessed the consistency of the entire scale with Cronbach’s alpha and the overall reliability of each factor of efficiency values. The reliability results in Table 3 show that Cronbach α values for all constructs are satisfactory, which indicates acceptable internal consistency.

It is important to note that composite reliability (CR) was used to determine reliability, and convergent validity was determined by checking the AVE against the square of the correlation coefficient between dimensions. The results indicate that the square root of the AVE of each construct was highest among the values of their latent variable coefficients. Likewise, discriminant validity was measured with a cross-loading matrix. As can be seen from Table 4, the items have a higher load value in their constructs, indicating the satisfactory discriminant validity of this property.

**Figure 2:**
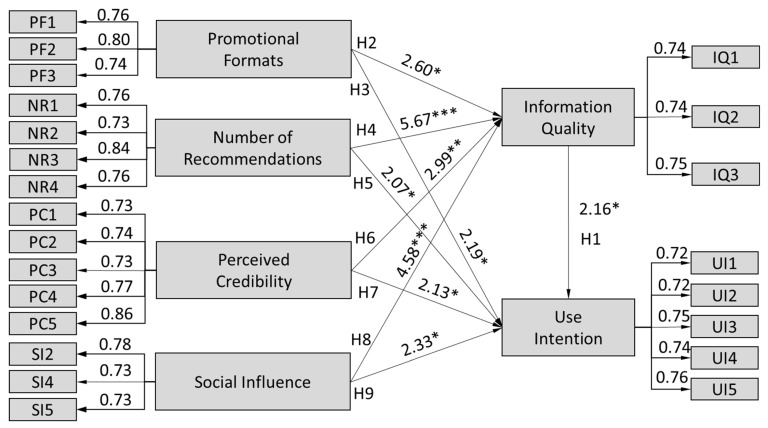
Results of path analysis. Notes: Significance of path coefficients: * *p* < 0.05, ** *p* < 0.01, *** *p* < 0.001.

Comparing the values obtained, an acceptable fit is found between the hypothesized model and the observed data (NFI = 0.95, NNFI = 0.95, CFI = 0.96, SRMR = 0.05). In addition, R^2^ and confidence intervals were employed to validate the structural paths of the conceptual model. The results in Figure 2 indicate that all hypotheses were supported, with a significance of *p* = 0.05.

Among the constructs, promotional format significantly affected information quality (H2: β = 0.18, *p* < 0.05) and intention to use telemedicine services (H3: β = 0.17, *p* < 0.05), and thus H2 and H3 were supported. In addition, the number of recommendations directly and positively influenced information quality (H4: β = 0.38, *p* < 0.001) and use intention (H5: β = 0.20, *p* < 0.05), and perceived credibility demonstrated a significant impact on Internet relationships (H6: β = 0.29, *p* < 0.01) and use intention (H7: β = 0.22, *p* < 0.05). Meanwhile, social influence significantly influenced Internet relationships (H8: β = 0.24, *p* < 0.001) and use intention (H9: β = 0.16, *p* < 0.05). The results also illustrate the involvement of Internet relationships in predicting the intention to use telemedicine services (H1: β = 0.33, *p* < 0.05). The results indicate that all hypothesized relationships were significant, and the hypothesized model is acceptable. 

## 5. Discussion

In this age of advanced networks, e-patients can access substantial information about healthcare offerings [75]. In order to respond to problems related to digital healthcare for potential patients and meet the growing need at the highest standards, digital marketing of telemedicine services is needed [75,76,77]. Digital healthcare marketing is a component of marketing that uses the Internet and networked technologies (mobile phones, desktop computers, and other devices) to promote telemedicine services.

The results of testing the first hypothesis suggest the relevance of quality information to the intention to use telemedicine services. Patients, considering the relevance and completeness of the information as the most important aspects before using telemedicine services, consult the online communities to which they have access. Additionally, telemedicine that produces efficient, specific, and pertinent information will affect users’ motivation to use these services. 

Based on the results of testing H2 and H3, it can be stated that the promotional format is an important marketing tool that can direct the feelings, perceptions, and behaviors of online patients. The results indicate that consumers who choose price reductions or promotional packages seek to have their decision to use telemedicine services influenced by social media. In conclusion, how different e-patients see the value of telemedicine services affects their plans to use them in different ways. 

Regarding promotional marketing, medical institutions can convey their product offerings directly on their websites. Website users can search for the specific information they want, and dialogue can be maintained between patients and the clinic as a result [40]. Medical facilities can assure communication with online patients by utilizing paid online advertising services, such as search engine marketing (SEM), which increases online visibility by using relevant keywords (this strategy typically also entails improving the site’s content to enhance exposure to search results for targeted keywords, or SEO), and (2) pay-per-click (PPC), which targets patients directly and provides a means of communicating with them directly.

At the same time, medical institutions can also choose to promote telemedicine services through social media or mobile platforms (applications), which is a direct-marketing technique. Direct marketing offers the possibility for medical institutions to educate and enlighten e-patients on telemedicine services, acquire patronage, and achieve the expected market share [41,42,43,44]. Pricing strategies for healthcare institutions must take into account costs and competition and e-patients’ perception of prices, determined by their knowledge of price as an indicator of quality and non-monetary prices [43,44]. Such a strategy can often be detrimental to online patients in forms other than higher service prices [48].

The findings regarding H4 and H5 suggest that e-patients prefer the opinions of virtual community members who post many messages sharing their experience [50,51,52]. Therefore, because online patients are influenced by volume recommendations, medical service companies should use such information, rather than sales volume, to present digital services. However, it should be noted that positive reviews have a significant impact on telemedicine services.

Another result from testing H6 and H7 is based on the credibility of telemedicine services. It was found that credibility had a positive impact on the use of healthcare services. Thus, along with the informational content, managers must also consider the credibility of online medical services provided. Marketers may reduce the risk of dissatisfaction with telemedicine services through other digital marketing activities. This can make digital marketing more successful.

Considering the social influence, the results of testing H8 and H9 demonstrate how members are brought together through online communities. The number of people with the same needs who are eager for information about telemedicine services has increased, and the dynamics of the transactional attitude of virtual community members have changed. Companies can also contact influencers to communicate with community members and improve e-patient loyalty and intent. Hence, influencers are an integral part of telemedicine services’ communication strategies. So far, they have been known as “opinion leaders”, but the digital transformation is not just about telehealth communication. It also means that emotional needs and a desire for guidance, for both healthy and sick people, are taken into account—and influencers are positioned to pass on credible and reliable health information.

On top of that, these results are consistent with the evidence reported by many authors [3,36,53,57,76], showing that the communication strategies developed by digital healthcare organizations are aimed at improving the quality of the telemedicine services they provide, and online patient education is the first course of action for this purpose [61]. However, this must be complemented by analyzing some essential elements of the business environment regarding barriers to entry, standards, and legislative elements concerning service policy [43]. The distribution can differ depending on the type of digital medical service; the social class, age, sex, and preferences of e-patients; and the peculiarities of the market.

Finally, these findings are in line with previous studies [17,25,48,74] showing that an understanding of the economic and managerial mechanisms of operations is required in order to make pertinent, real, and especially opportune decisions that could counteract turbulent environmental threats and increase the potential for telemedicine service companies. Developing technologies for optimizing the patient experience and content and providing personalized content for effective branding are the explicit goals of digital healthcare providers.

Although this research has several findings, it should be kept in mind that this was an online experiment with numerous limitations. First, the findings of this study and the inferences that may be made from them are only applicable to those living in Romania. In order to investigate the cultural influences of social media, future research should examine the behavioral intentions of patients in other countries. Second, other than the constructs used in this study, there may be other variables that could influence the use of telemedicine services. Future studies could explore other relevant variables (e.g., personality traits, telehealth knowledge) in order to better understand the demands of social media users. Furthermore, operators of community websites could segment e-patients who use medical services in order to more closely meet their needs. 

## 6. Conclusions

In the current context, we are discussing the variety of analog and digital contexts that characterize the realities of life and change in ever-faster cycles. Radically thought out from a human point of view, this is not necessarily an enrichment at first sight. Humans are actually creatures of habit. Especially in the telemedicine services segment, there is a growing number of approaches for digitally communicating the best solutions to the relevant target groups in the given contexts.

With increasing agility, the digitization and the discoveries of psychological research are not mutually exclusive but complement each other perfectly: patients behind smartphones are not only made visible by qualitative research using algorithms and numbers, but also benefit from a deeper understanding of their needs, desires, motives, fears, and barriers through digital implementation.

These days, digital healthcare companies focus their attention on e-patients by providing high-quality telemedicine services at reasonable prices, using digital health marketing as a tool. For this reason, by placing the e-patient at the center of these concerns, digital marketing has become a function of the activity of telehealth organizations. Whatever the purpose, digital healthcare organizations operate under the influence of complex, ever-changing digital marketing strategies that make telehealth unable to function as a typical competitive market.

In the present study, the authors demonstrate that knowing the purpose for telemedicine service use in Romania can enable virtual community sites to deliver online services that more closely meet members’ needs and enable members to consistently satisfy their own requirements through virtual communities. Moreover, the anticipated linear trend will have substantial financial and economic consequences.

From a theoretical standpoint, this study combines the TAM and UTAUT models with the IS success model to explain users’ continual telemedicine usage intentions as a consequence of gaining information from virtual communities. Consequently, it contributes considerably to the existing studies that examine the impact of online communities on the intention to use telemedicine services.

Taking into account the practical implications, this research offers several suggestions for managers and marketers to apply digital marketing. The first suggestion is that managers should pay attention to information quality. In general, most patients want to obtain more information from websites or social networking platforms. The second suggestion is that they might use more abstract descriptions, because that could have a positive influence on the use of telemedicine services.

Research among health organizations showed that their use of digital marketing, although considered useful, is insufficiently developed in Romania, with certain marketing methods and techniques being applied only by private organizations due to the level of competition in the market in which they operate. On top of that, this study does not exhaust the topic but could be an approach that addresses the complexity of changing digital marketing concepts in an increasingly interconnected world. As this study demonstrates, measuring the impact of digital marketing in telemedicine services is a global issue that decision makers in organizations are increasingly focusing on.

## Figures and Tables

**Table 1:** Measurement items and descriptive statistics for all variables.

Construct	Items
Promotional format (PF) [49,50,51,52,53]	(PF1) The promotional packages for telemedicine services keep their commitments.
	(PF2) The discount prices offered for telemedicine services are attractive.
	(PF3) I think promotional packages for telemedicine services solve my healthcare problems.
Number of recommendations (NR) [54,55,56,57,58]	(NR1) I follow a number of recommendations when I choose to use telemedicine services.
	(NR2) I think the number of recommendations provides accurate information about telemedicine services.
	(NR3) I prefer to use telemedicine services if they are recommended.
	(NR4) I would promote telemedicine services to others.
Perceived credibility (PC) [59,60,61,62,63,64,65,66]	(PC1) It is safe for me to use telemedicine services.
	(PC2) I believe telemedicine services will protect my privacy.
	(PC3) I have heard people talking about this issue.
	(PC4) I think telemedicine services are trustworthy.
	(PC5) I have heard people talking about this particular telemedicine service.
Social influence (SI) [67,68,69,70,71,72,74]	(SI1) People who influence me use telemedicine services.
	(SI2) Comments on social media regarding telemedicine services make me convinced.
	(SI3) I like to discuss details about using telemedicine services with a friend or in my friend network.
Information quality (IQ) [36,37,38,39,40,41]	(IQ1) The website information is in accordance with my healthcare needs.
	(IQ2) I expand my individual interpersonal relationships by following online information about telemedicine services.
	(IQ3) I believe that websites provide complete and up-to-date information on medical services.
Use intention (UI) [42,43,44,45,46,47,48]	(UI1) I will continue to use telemedicine services in the future after analyzing the feedback.
	(UI2) I intend to check the quality of information of telemedicine services in the near future.
	(UI3) I have a high willingness to use telemedicine services.
	(UI4) I might consider using telemedicine services in the future after analyzing social media information.
	(UI5) I use promotional packages related to telemedicine services as often as possible.

**Table 2:** Sample characteristics.

		Frequency	Percentage (%)
Gender	Male	186	42.08
	Female	256	57.92
	Total	442	100
Age (years)	18–25	141	31.90
	26–35	115	26.02
	36–50	98	22.17
	Above 50	88	19.91
	Total	442	100

**Table 3:** Summary of measurement scales.

Variable	Construct Items	Standard Deviation	Factor Loading	Cronbach’s α	Composite Reliability	AVE
Promotional format (PF)	PF 1	0.92	0.76	0.82	0.82	0.72
	PF 2	0.99	0.80			
	PF 3	0.94	0.74			
Number of recommendations (NR)	NR 1	0.78	0.76	0.88	0.87	0.76
	NR 2	0.97	0.73			
	NR 3	0.99	0.84			
	NR 4	0.96	0.76			
Perceived credibility (PC)	PC 1	0.99	0.73	0.88	0.89	0.73
	PC 2	0.98	0.74			
	PC 3	0.78	0.73			
	PC 4	0.94	0.77			
	PC 5	0.96	0.86			
Social influence (SF)	SI 1	0.92	0.78	0.77	0.80	0.73
	SI 2	0.94	0.73			
	SI 3	0.92	0.73			
Information quality (IQ)	IQ 1	0.92	0.74	0.79	0.79	0.72
	IQ 2	0.96	0.74			
	IQ 3	0.91	0.75			
Use intention (UI)	UI 1	0.96	0.72	0.87	0.87	0.71
	UI 2	0.91	0.73			
	UI 3	0.79	0.75			
	UI 4	0.99	0.74			
	UI 5	0.96	0.76			

**Table 4:** Correlation matrix of variables.

	Promotional Format	Number of Recommendations	Perceived Credibility	Social Influence	Information Quality	Use Intention
Promotional format	0.76					
Number of recommendations	0.41	0.78				
Perceived credibility	0.36	0.36	0.76			
Social influence	0.38	0.37	0.34	0.75		
Information quality	0.36	0.34	0.32	0.34	0.74	
Use intention	0.31	0.29	0.28	0.28	0.26	0.73

## Data Availability

Not applicable.
